# Leaf polyphenol profile and SSR-based fingerprinting of new segregant *Cynara cardunculus* genotypes

**DOI:** 10.3389/fpls.2014.00800

**Published:** 2015-01-21

**Authors:** Gaetano Pandino, Sara Lombardo, Andrea Moglia, Ezio Portis, Sergio Lanteri, Giovanni Mauromicale

**Affiliations:** ^1^Dipartimento di Agricoltura, Alimentazione e Ambiente Università degli Studi di CataniaCatania, Italy; ^2^Dipartimento di Scienze Agrarie, Forestali e Alimentari, Università degli Studi di TorinoGrugliasco, Italy

**Keywords:** *Cynara cardunculus*, genotype, growing season, SSRs analysis, caffeoylquinic acids, flavones

## Abstract

The dietary value of many plant polyphenols lies in the protection given against degenerative pathologies. Their *in planta* role is associated with the host's defense response against biotic and abiotic stress. The polyphenol content of a given plant tissue is strongly influenced by the growing environment, but is also genetically determined. Plants belonging to the *Cynara cardunculus* species (globe artichoke and the cultivated and wild cardoon) accumulate substantial quantities of polyphenols mainly mono and di-caffeoylquinic acid (CQA) in their foliage. Transgressive segregation for CQA content in an F_1_ population bred from a cross between a globe artichoke and a cultivated cardoon led to the selection of eight segregants which accumulated more CQA in their leaves than did those of either of their parental genotypes. The selections were grown over two seasons to assess their polyphenol profile (CQAs, apigenin and luteolin derivatives and narirutin), and were also fingerprinted using a set of 217 microsatellite markers. The growing environment exerted a strong effect on polyphenol content, but two of the selections were able to accumulate up to an order of magnitude more CQA than either parent in both growing seasons. Since the species is readily vegetatively propagable, such genotypes can be straightforwardly exploited as a source of pharmaceutically valuable compounds, while their SSR-based fingerprinting will allow the genetic identity of clonally propagated material to be easily verified.

## Introduction

Polyphenols are a diverse group of plant secondary metabolites involved in both protection against biotic and abiotic stresses, and in plant growth and reproduction (Friedman, 1997; Bravo, 1998). They represent an important component of the human diet, as many epidemiological studies have demonstrated that their consumption can be related to a decreased incidence or severity of a number of chronic diseases (Jang et al., 1997; Arai et al., 2000; Birt et al., 2001; Miller and Snyder, 2012). This class of molecule is synthesized by the phenylpropanoid pathway, the initial step of which comprises the deamination of L -phenylalanine to form trans-cinnamic acid, a reaction catalyzed by phenylalanine ammonium lyase (PAL). PAL activity (as well as that of other enzymes in the phenylpropanoid pathway) is influenced by a number of environmental factors, both during the plant's growth, the post-harvest storage of its products, and their processing into food or other products (Beckman, 2000; Tomás-Barberán and Espín, 2001; Kalt, 2005; Pandino et al., 2012, 2013a; Ezekiel et al., 2013). However, genotype is also a major determinant of variation in polyphenol content and profile (Mpofu et al., 2006; Farshadfar et al., 2012; Alfaro et al., 2013; Gündüz and Özdemir, 2014).

The *Cynara cardunculus* complex includes two cultivated taxa [the globe artichoke (var. *scolymus*) and the cultivated cardoon (var. *altilis*)], along with their progenitor, the wild cardoon (var. *sylvestris*). The wild taxon is distributed across the western and central part of the Mediterranean Basin, the Canary Islands and Madeira and in the Americas; var. *altilis* is a minor crop cultivated mainly in southern Europe (Portis et al., 2005), while var. *scolymus* is cropped quite extensively both in Mediterranean countries and has recently spread to Americas and to China (http://faostat.fao.org/, 2012). Crosses between members of the three taxa are viable, fertile and highly variable at the phenotypic level (Lanteri et al., 2006). While the major product of the globe artichoke is the edible head, the plants' leaves have been shown to represent a potentially productive source of flavones and caffeoylquinic acids (CQAs), which have various industrial, pharmaceutical and cosmetic applications (Wang et al., 2003; Pinelli et al., 2007; Moglia et al., 2008, 2009; Comino et al., 2009; Lattanzio et al., 2009). Analyses of cloned individuals selected from the autochthonous Sicilian globe artichoke varieties “Violetto di Sicilia” and “Spinoso di Palermo” have shown that leaf polyphenol content is a highly variable trait (Pandino et al., 2011, 2013b,c). Since the first linkage map produced for globe artichoke (Lanteri et al., 2006), a number of other segregating populations have been exploited for genetic mapping (Portis et al., 2012; Martin et al., 2013), based on different classes of molecular markers (Acquadro et al., 2005, 2009; Cravero et al., 2005, 2007). The resulting maps were used to identify and locate the major loci controlling the key agronomic traits. Here, the intention was to use a set of segregating progeny derived from a var. *scolymus* × var. *altilis* cross to characterize segregation for leaf polyphenol content, as well as assigning their genotype with respect to a large panel of SSR (microsatellite) loci.

## Materials and methods

### Experimental plots and sampling

The experimental field at the University of Catania, Sicily (37°25′N; 15°30′E; 10 m a.s.l.) lies in an area representative of commercial var. *scolymus* cultivation. The local climate consists of mild and wet winters and hot, dry summers. A segregating population of 94 F_1_ hybrids, previously described by Portis et al. (2009), along with six clonally propagated plants of each of the var. *scolymus* genotype “Romanesco C3” (female parent) and the var. *altilis* genotype “Altilis 41” (male parent), were grown over the 2009–2010 season (hereafter termed “2010”). “Romanesco C3” is late maturing plant, which produces large purple-green heads, while “Altilis 41” was selected for its high biomass yield potential. Following a preliminary screening of leaf polyphenol content, eight transgressive (with respect to CQA production) F_1_ progeny were clonally reproduced from 12 month old plants by transplanting their semi-dormant offshoots (“ovoli”). For each of the eight selected transgressive genotypes, 15 clonally propagated plants were obtained and grown over the 2010–2011 season (hereafter “2011”) in plots arranged in the form of a randomized block design with three replications. In early November in both seasons, at a time when each plant bore at least 30 leaves, a sample of about 10 disease-free leaves per replicate per F_1_ individual was harvested and washed in tap water. The leaf material was chopped and blended in a domestic food processor, then freeze-dried, divided into three aliquots and stored at −20°C pending HPLC analysis.

### HPLC-based screening of caffeoylquinic acids in the F_1_ progeny

A 50 mg sample of freeze-dried leaf tissue from each of the 94 F_1_ hybrid plants was suspended in 1.95 mL 70:30 (v/v) methanol: water and sonicated for 20 min in a water bath. After centrifugation (10,000 × g for 10 min), a 10 μL aliquot of the supernatant, derived by filtration through a 0.45 μm Anotop 10 filter (Whatman, Maidstone, UK), was injected into an LC-920 device (Varian, Palo Alto, California, USA). The CQA content of the sample was quantified by means of reverse-phase HPLC, using an analytical Luna C18 column (2 × 150 mm, particle size 3 μm, 100 Å; Phenomenex, Torrance, California, USA), along with a 2 × 4 mm pre-column (Phenomenex), as described by Menin et al. (2010). The mobile phase was a 1:1000 (v/v) mixture of de-gassed glacial acetic acid and ultrapure water (eluant A) and a 1:1000 (v/v) mixture of acetic acid and acetonitrile (eluant B). The initial elution solvent was 5% B, 95% A, increasing linearly to 35% B, 65% A over 28 min. The flow rate was 0.5 mL min^−1^ and the eluate was monitored spectrophotometrically at 300 nm and 330 nm. Molar quantification of mono-and di-CQAs was based on pre-established calibration curves for both 5-*O*-CQA and 1,5-*O*-diCQA.

### HPLC-based characterization of polyphenols profile in the selected F_1_ genotypes

Polyphenols were then extracted from the selected eight F_1_ progeny as described by Pandino et al. (2010), and subjected to HPLC analysis using a series 1200 instrument (Agilent Technologies, Palo Alto, CA, USA) equipped with ChemStation software (B.03.01) and a diode array detection system. Separations were achieved by passing the samples through a Zorbax Eclipse XDB-C18 column (4.6 × 150 mm; 5.0 μm particle size), operated at 30°C, with a 0.2 μm stainless steel inline filter. The protocol was adapted from Pandino et al. (2010): the mobile phase comprised 1: 1000 (v/v) mixture of formic acid: water (solvent A) and 1: 1000 (v/v) mixture of formic acid: acetonitrile (solvent B), using a flow rate of 0.5 mL min^−1^. The process began with a mixture of 5% B, 95% A, reaching 10% B after 10 min, 40% B after 30 min, and keeping constant at 90% B from 50 to 58 min. Chromatograms were recorded at 280, 310, and 350 nm and the data collected between 200 and 600 nm. Compound identification was based on retention time, UV spectrum, and with reference to compounds identified in var. *scolymus* by Wang et al. (2003) and Schütz et al. (2004). Molar quantification of each compound was based on calibration curves generated from available standards. CQAs are presented according to the recommended IUPAC numbering system. Apigenin and luteolin conjugates were quantified as apigenin-7-*O*-glucoside and luteolin-7-*O*-glucoside, respectively. Each data point represented the means of three independent experiments. Polyphenol contents are expressed as g kg^−1^ dry matter (DM).

All reagents and solvents (analytical or HPLC grade) were purchased from VWR (Leighton Buzzard, UK). Apigenin-7-*O*-glucoside, apigenin, luteolin-7-*O*-glucoside, luteolin, 5-*O*-CQA (chlorogenic acid), hesperetin from Extrasynthese (Lyon, France), cynarin (1,3-di-*O*-CQA) from Roth (Karlsruhe, Germany). A Milli-Q system (Millipore Corp., Bedford, MA, USA) was used to provide ultrapure water.

To evaluate the dry matter (DM) content in selected genotypes, a 100 g sample of fresh leaf tissue was oven-dried at 65°C (Binder, Milan, Italy) until a constant weight had been reached, then re-weighed. Results are expressed as % of dry matter on fresh weight.

### SSR fingerprinting

DNA was extracted from young leaves of “Romanesco C3” and “Altilis 41” and the eight selected F_1_ progeny following the Lanteri et al. (2004) protocol, then used as template for amplification with a set of 217 SSR primer pairs (Acquadro et al., 2005, 2009; Scaglione et al., 2009) which recognize loci distributed over all 17 *C. cardunculus* chromosomes (Portis et al., 2012, 2014). Each 10 μL PCR was based on 7 ng template combined with 1x PCR buffer, 1 mM MgCl_2_, 0.5 U Taq DNA polymerase (Qiagen Inc., Venlo, Netherlands), 40 nM 5′-labeled (FAM, HEX, or TAMRA) forward primer, 40 nM unlabeled reverse primer and 0.2 mM dNTP. A touchdown cycling regime was applied, consisting of an initial denaturation of 94°C/2.5 min, followed by nine cycles of 94°C/30 s, 63°C/30 s (decreasing by 0.7°C per cycle), 72°C/60 s, and 30 further cycles of 94°C/30 s, 57°C/30 s, 72°C/60 s. Where only weak amplification was achieved, the MgCl_2_ concentration was raised to 1.5 mM and the final annealing temperature lowered to 55°C. The amplicons were separated on an ABI3730 capillary DNA sequencer (Applied Biosystem Inc., Foster City, CA, USA). Internal ROX-labeled GS500 size standards were included in each capillary. The SSR data were collected by GeneMapper v3.5 software (Applied Biosystems) and analyzed using the GenAlex Excel package (Peakall and Smouse, 2006). A co-phenetic distance matrix was generated as described by Smouse and Peakall (1999) and used to construct a UPGMA-based dendrogram (Sneath and Sokal, 1973) implemented within the NTSYS software package v2.10 (Rohlf, 1998). The minimum number of SSR loci needed to fully discriminate all individuals was searched within the set of nine most informative SSRs previously identified for the “Romanesco C3” × “Altilis 41” progeny by Lanteri et al. (2012), on the basis of their distribution in different linkage groups.

### Statistical analysis

Population means, standard deviations, distribution histograms were calculated using SPSS statistical software. The data were subjected to a One-Way analysis of variance (ANOVA), and means were separated from one another using Tukey's HSD or Fisher's LSD (least significant difference) test. A Mantel (1967) test was performed to establish correlations between the similarity matrices generated by a SSR subset and the one generated using the complete set of 217 SSRs.

## Results and discussion

### Variation in caffeoylquinic acid content in the full F_1_ population

**Figure 1:**
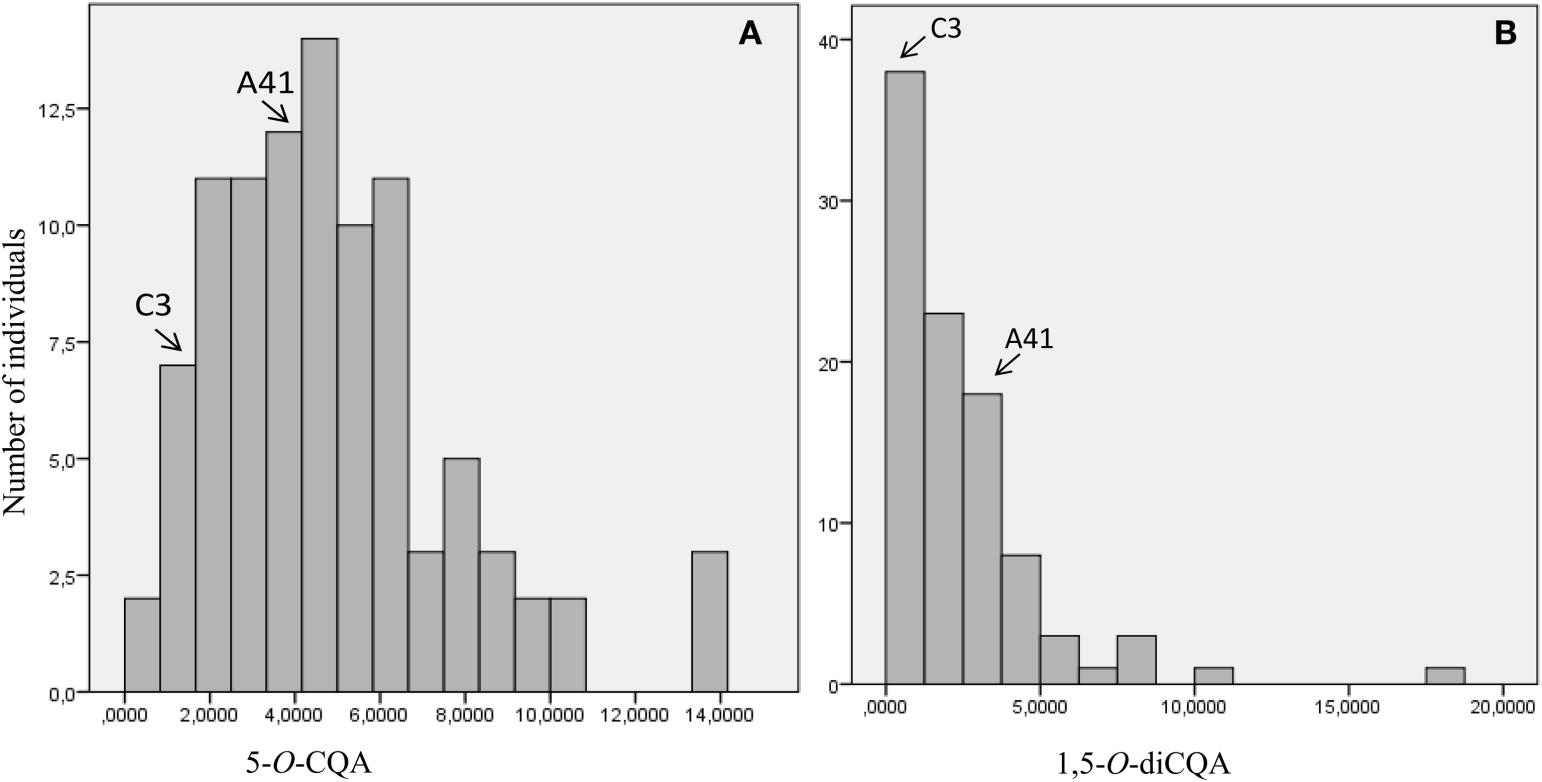
**Frequency distribution of CQA content in the F_1_ population derived from the cross “Romanesco C3” × “Altilis 41.” (A)** 5-*O*-CQA, **(B)** 1,5-*O*-diCQA. The vertical axis indicates the number of individuals per trait value class and the horizontal axis the trait value class. Parental genotypes (“C3”: “Romanesco C3,” A41: “Altilis 41”) are indicated by arrows.

Analysis of the methanolic extracts showed that the major leaf phenolic compounds present were chlorogenic acid (5-*O*-caffeoylquinic acid; 5-*O*-CQA) and 1,5-*O*-dicaffeoylquinic acid (1,5-*O*-diCQA). The content of both these polyphenols contrasted between “Romanesco C3” and “Altilis 41,” with the latter containing more of both 5-*O*-CQA and 1,5-*O*-diCQA (1.71 and 1.30 g kg-1 DM, respectively) than the former (0.61 and 0.16 g kg^−1^ DM, respectively) (Table 1). Both the 5-*O*-CQA and 1,5-*O*-diCQA content varied continuously across the full F_1_ population (Figure 1), implying polygenic inheritance for both CQAs. A selection of eight of the F_1_ progeny was made on the basis that they harbored a higher CQA content than either parental genotype, as a result of transgressive segregation derived from additive gene action. Similar segregation behavior for 5-*O*-CQA content has been noted in a population derived from the cross *Jacobaea vulgaris* × *Jacobaea aquatica* (Kirk et al., 2012) as well as in maize (Bushman et al., 2002).

**Table 1:** **CQA content (g kg^−1^ DM) of the parental genotypes “C3” (“Romanesco C3”), “A41” (“Altilis 41”) and their F_1_ progeny**.

**Metabolites**	**Parental genotypes**			**F_1_ population**		
	**C3**	**A41**	***p*-value**	**Mean**	**Range**	***SE***
5-*O*-caffeoylquinic acid	0.61 ± 0.13	1.71 ± 0.32	*p* ≤ 0.05	1.92	0.18–5.72	0.11
1,5-*O*-dicaffeoylquinic acid	0.16 ± 0.05	1.30 ± 0.19	*p* ≤ 0.05	0.95	0.12–7.36	0.10

### Seasonal variation for polyphenol profile and DM content

**Figure 2:**
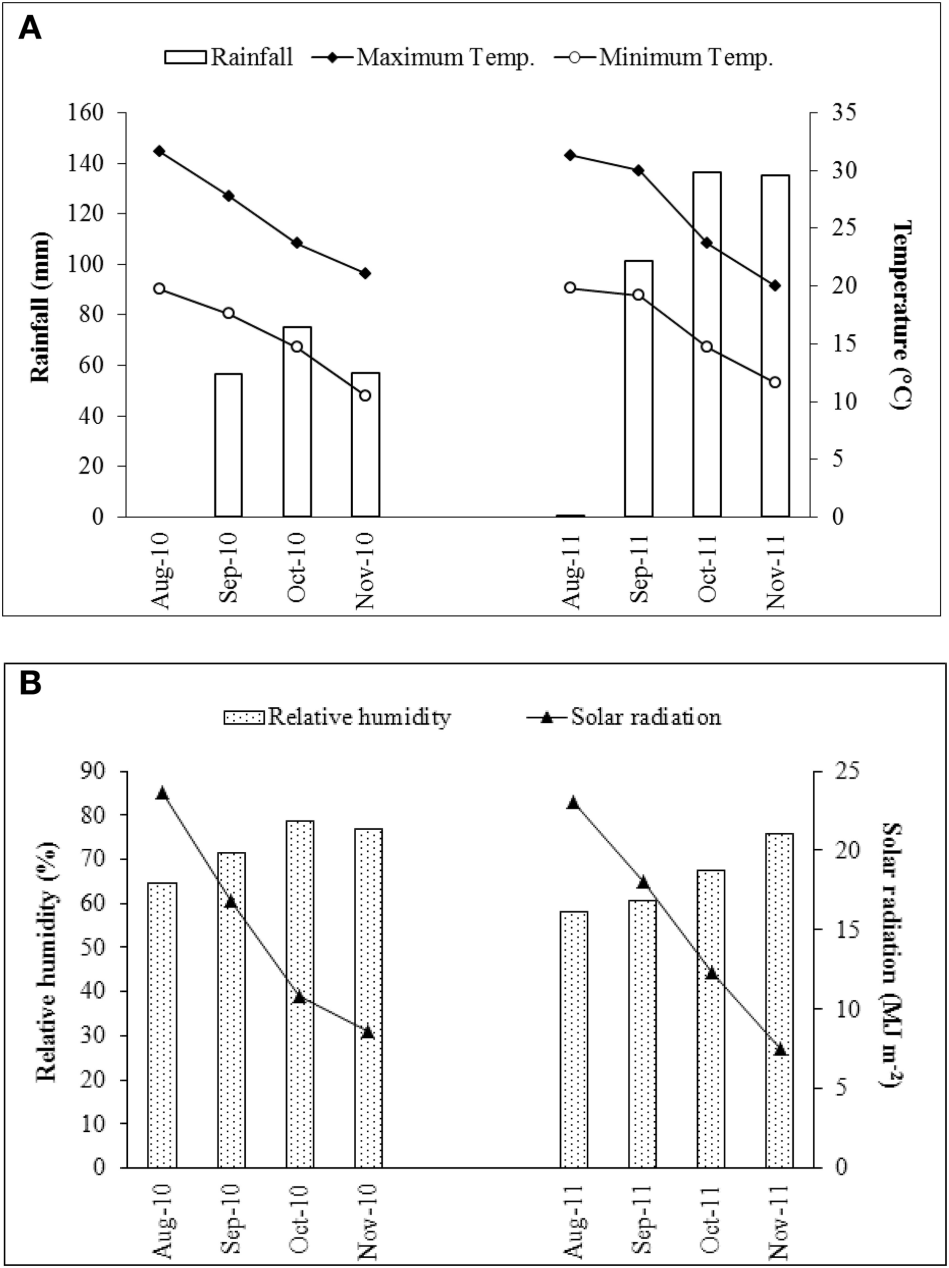
**Variation in climatic parameters between the 2010 and 2011 seasons**. **(A)** Rainfall and mean air temperature, **(B)** relative humidity and solar radiation.

As shown in the Table 2, large differences in the content of both total measured polyphenols (TMP) and DM were noted between the 2010 and 2011 seasons. In 2010, TMP ranged from 6.86 to 36.20 g kg^−1^ DM, whereas in 2011 the extremes were 5.12 and 19.47 g kg^−1^ DM; while DM fluctuated from 15 to 24% in 2010, but only from 11 to 16% in 2011. The content of luteolin was also strongly season-dependent (2.15–14.75 g kg^−1^ DM in 2010 and 2.26–8.29 g kg^−1^ DM in 2011); the coefficient of variation (CV) for this trait was 39% (Table 3). An even higher CV (93%) was noted for the content of apigenin derivatives followed by total content of caffeoylquinic acids and narirutin (CV of 50 and 52%, respectively) (Table 3). This major season-to-season variability probably reflected considerable differences in the prevailing climatic conditions (Figure 2). Precipitation was 189 mm in 2010, but 373 mm in 2011 as a result of rainfall heavily concentrated in a short period of time; despite this, 2010 experienced overall a higher average relative humidity. Air temperature, water availability, relative humidity, and accumulated solar radiation are all known to affect the polyphenol content of a number of fruits and vegetables (Connor et al., 2002; Dumas et al., 2003). The most important environmental factors responsible for the non-genetic determination of polyphenol content in potato are the quantity of rainfall and the relative humidity (Lombardo et al., 2013). In sour jujube fruits, a negative correlation has been established between the volume of annual precipitation and polyphenol content (Sun et al., 2011), while water shortage promotes the accumulation of phenolics in olive (Martinelli et al., 2012). In contrast, a positive correlation has been suggested between precipitation and polyphenol content in both the virgin oil made from fruit of the olive variety “Chétoui” (Temine et al., 2006) and in murtilla fruit (Alfaro et al., 2013). Little is known of the mechanistic basis of how water availability affects secondary metabolite content (Ramakrishna and Ravishankar, 2011), although it is generally assumed that abiotic stress acts to promote polyphenol synthesis (Dixon and Paiva, 1995).

**Table 2:** **Total TMP content (g kg^−1^ DM) and DM (%) of the leaves of plants grown in both 2010 and 2011**.

**Genotype**	**TMP^(a)^**			**Dry matter**		
	**2010**	**2011**	**Mean**	**2010**	**2011**	**Mean**
*Altilis 41*	16.97	5.12	11.04^g^	23	14	18^ab^
*Romanesco C3*	6.86	5.47	6.16^h^	15	11	13^e^
1	22.19	6.58	14.38^f^	16	14	15^d^
12	26.52	7.68	17.10^e^	19	15	17^bd^
35	25.23	6.24	15.73^ef^	20	16	18^ac^
48	32.41	13.91	23.16^bc^	24	15	19^a^
69	29.97	13.72	21.84^c^	20	12	16^cd^
72	29.71	9.78	19.74^d^	22	14	18^ac^
74	33.42	14.90	24.16^b^	23	12	18^ac^
78	36.20	19.47	27.83^a^	24	11	17^ac^
Mean	25.95^a^	10.26^b^		20^a^	13^b^	
LSD interaction_(*P* ≤ 0.05)_	2.54			2.90		
CV^(b)^ (%)	40			12		

*Different letters associated with the set of means indicate significance based on Fisher's protected LSD test (P ≤ 0.05)*.

(a) *TMP, total measured polyphenols*.

(b) *CV, coefficient of variation*.

**Table 3:** **The content (g kg^−1^ DM) of CQAs, apigenin derivatives, luteolin derivatives and narirutin in the leaves of plants grown in both 2010 and 2011**.

**Genotype**	**Tot CQA^(a)^**			**Tot API^(b)^**			**Tot LUT^(c)^**			**Tot NAR^(d)^**		
	**2010**	**2011**	**Mean**	**2010**	**2011**	**Mean**	**2010**	**2011**	**Mean**			
*Altilis 41*	1.86	1.08	1.47^d^	12.96	1.55	7.25^a^	2.15	2.26	2.20^f^	–	0.24	0.12^e^
*Romanesco C3*	0.79	0.44	0.61^d^	0.19	0.94	0.56^f^	5.34	4.09	4.71^e^	0.54	trace	0.27^c^
1	15.85	0.46	8.15^c^	1.08	1.22	1.15^e^	5.26	4.64	4.95^e^	–	0.25	0.12^e^
12	14.46	1.09	7.77^c^	4.61	0.62	2.61^d^	6.94	5.97	6.45^d^	0.51	–	0.25^c^
35	14.92	1.93	8.42^c^	0.66	trace	0.33^f^	9.09	4.31	6.70^d^	0.56	trace	0.28^c^
48	18.13	6.23	12.18^a^	–	1.15	0.57^f^	14.28	6.19	10.23^a^	–	0.34	0.17^d^
69	13.40	5.52	9.46^b^	1.24	1.55	1.39^e^	14.75	6.27	10.51^a^	0.58	0.39	0.48^a^
72	15.23	3.68	9.45^b^	4.39	0.75	2.57^d^	10.09	5.09	7.59^c^	–	0.26	0.13^de^
74	14.91	4.94	9.92^b^	4.54	1.37	2.95^c^	13.32	8.29	10.80^a^	0.65	0.31	0.48^a^
78	14.49	11.00	12.74^a^	10.85	0.80	5.82^b^	10.15	7.67	8.91^b^	0.71	–	0.35^b^
Mean	12.41^a^	3.64^b^		4.05^a^	0.99^b^		9.13^a^	5.48^b^		0.35^a^	0.18^b^	
LSD interaction_(*P* ≤ 0.05)_	1.47			0.45			0.95			0.06		
CV^(e)^ (%)	50			93			39			52		

*Different letters associated with the set of means indicate significance based on Fisher's protected LSD test (P ≤ 0.05)*.

(a) *Tot CQA, total content of caffeoylquinic acids*.

(b) *Tot API, total content of apigenin and its derivarives*.

(c) *Tot LUT, total content of luteolin and its derivarives*.

(d) *Tot NAR, total content of narirutin*.

(e) *CV, coefficient of variation*.

The variation in both TMP and DM content was much greater between the two seasons than between the genotypes, and the genotype x season interaction was also highly significant (Table 4). The environment on its own explained much of the variation in the content of CQA, TMP and DM, as well as those of apigenin and lutein derivatives and narirutin. A similar experience has been reported by Lombardo et al. (2009), who noted extensive season-to-season variation in TMP content in the heads of a number of var. *scolymus* varieties, as well as by Zheng et al. (2012) working with *Ribes* sp. fruit.

**Table 4:** **Mean square as absolute value and percentage of total (in brackets) of effects resulting from analysis of variance**.

	**Source of variation**		
	**Genotype (G)**	**Season (S)**	**G × S**
Degree of freedom	9	1	9
CQA^a^	97^***^ (8)^g^	1154^***^ (89)	44^***^ (3)
API^b^	33^***^ (16)	140^***^ (69)	30^***^ (15)
LUT^c^	49^***^ (19)	200^***^ (76)	14^***^ (5)
NAR^d^	0.1^***^ (8)	0.5^***^ (74)	0.2^***^ (18)
MP^e^	258^***^ (7)	3678^***^ (92)	46^***^ (1)
DM^f^	15^***^ (3)	504^***^ (94)	14^***^ (3)

a *CQA, total content of caffeoylquinic acids*.

b *API, total content of apigenin and its derivatives*.

c *LUT, total content of luteolin and its derivatives*.

d *NAR, total content of narirutin*.

e *MP, total content of measured polyphenols*.

f *DM, dry matter content*.

g*** *, Indicate significant at P ≤ 0.001*.

### Polyphenols in the high CQA selections

**Figure 3:**
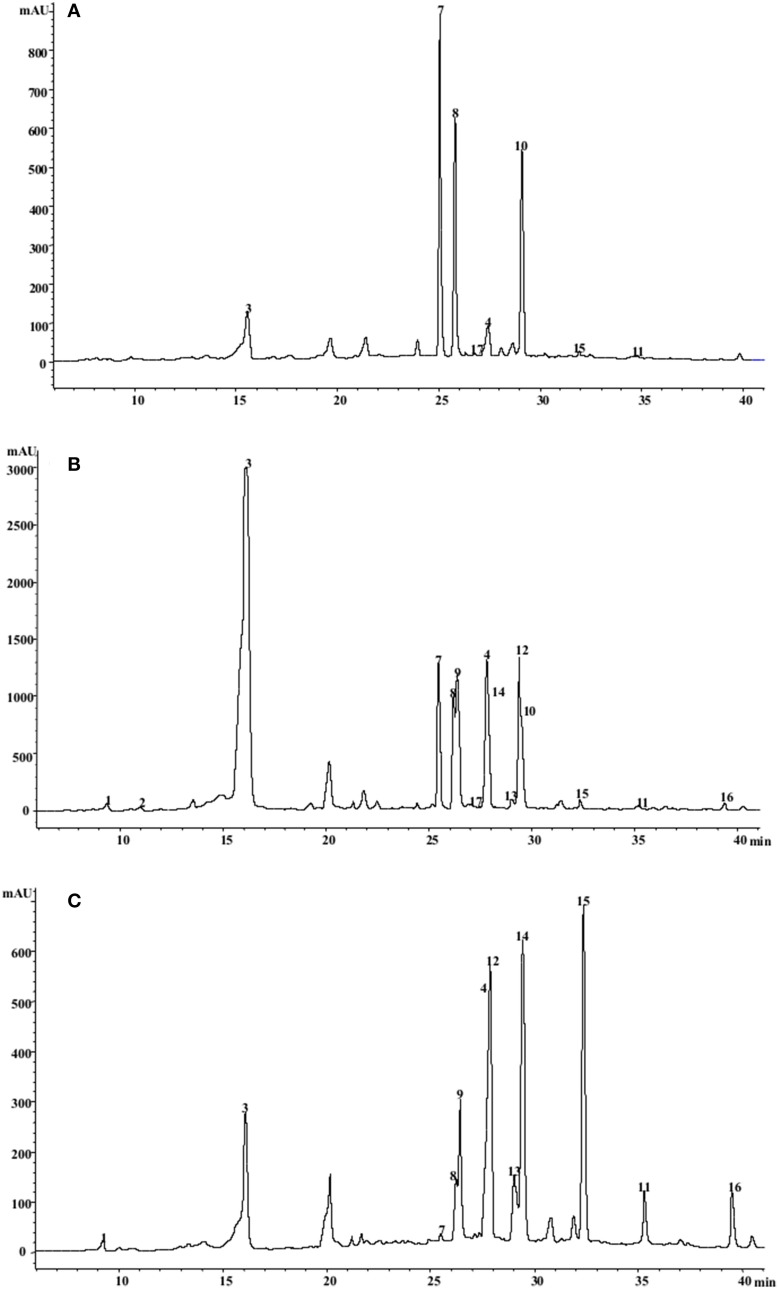
**HPLC/DAD chromatograms of *C. cardunculus* leaves acquired at 310 nm**. **(A)** “Romanesco C3,” **(B)** “Selection 78,” **(C)** “Altilis 41.” For peak assignment see Tables 5, 6.

The CQAs contained in larger amounts in both the parents and F1 individuals were the 5-*O*-CQA and the 1,5-*O*-di CQAs, which were the only ones always detectable in the selections over the two growing seasons (Tables 5, 6). Among the selected F_1_ individuals, selection 78 produced the most 5-*O*-CQA in both 2010 and 2011 (13.16 and 7.28 g kg^−1^ DM, respectively) (Figure 3), followed by selection 35 in 2010 (12.34 g kg^−1^ DM) and selection 69 in 2011 (4.53 g kg^−1^ DM) (Tables 5, 6). Selection 1 produced the most 1,5-*O*-di CQA in 2010 (9.48 g kg^−1^ DM), followed by selection 48 in the same season (6.64 g kg^−1^ DM).

**Table 5:** **Phenolic content (g kg^−1^ DM) of the leaves of plants grown in 2010**.

**No**.	**Compound**	**Genotype**									
		**Altilis 41**	**Romanesco C_3_**	**1**	**12**	**35**	**48**	**69**	**72**	**74**	**78**
1	1-*O*-caffeoylquinic acid	0.09^c^	nd^(b)^	0.19^c^	0.62 ± 0.1^b^	0.16^c^	0.89 ± 0.1^a^	0.83 ± 0.1^a^	0.84 ± 0.1^a^	0.99 ± 0.1^a^	0.20^c^
2	3-*O*-caffeoylquinic acid	nd	nd	nd	nd	0.23^a^	nd	trace	trace	nd	0.16^b^
3	5-*O*-caffeoylquinic acid	1.18 ± 0.1^e^	0.57^e^	5.56 ± 0.4^d^	8.26 ± 1.0^bc^	12.34 ± 1.2^a^	9.33 ± 0.7^b^	6.76 ± 0.2^cd^	8.05 ± 0.9^bc^	7.82 ± 0.9^bc^	13.16 ± 1.6^a^
4	1,5-*O*-dicaffeoylquinic acid	0.59 ± 0.1^ef^	0.22^f^	9.48 ± 0.4^a^	5.58 ± 0.8^c^	2.19 ± 0.1^d^	6.64 ± 0.6^b^	5.52 ± 0.2^c^	6.04 ± 0.5^bc^	6.10 ± 0.6^bc^	0.97 ± 0.1^e^
5	monosuccinyldicaffeoylquinic acid	nd	nd	0.36^b^	nd	nd	0.78 ± 0.1^a^	nd	nd	nd	nd
6	4,5-*O*-dicaffeoylquinic acid	nd	nd	0.26^b^	nd	nd	0.49 ± 0.1^a^	0.29^b^	0.30^b^	nd	nd
7	Luteolin 7-*O*-rutinoside	0.07^g^	2.25^e^	nd	nd	0.17^fg^	3.50 ± 0.1^c^	4.18 ± 0.2^b^	0.30^f^	4.76 ± 0.1^a^	2.96 ± 0.2^d^
8	Luteolin 7-*O*-glucoside	0.34^e^	1.67^d^	3.07 ± 0.4^c^	4.31 ± 0.5^a^	3.75 ± 0.2^b^	2.10 ± 0.1^d^	2.85 ± 0.1^c^	1.87 ± 0.3^d^	4.35 ± 0.1^a^	1.96 ± 0.2^d^
9	Luteolin 7-*O*-glucuronide	1.01 ± 0.1^f^	nd	nd	nd	1.99 ± 0.2^e^	5.13 ± 0.1^a^	4.28 ± 0.2^b^	2.73 ± 0.2^d^	nd	3.42 ± 0.5^c^
10	Luteolin 7-*O*-malonylglucoside	nd	1.36^e^	1.78 ± 0.2^d^	2.63 ± 0.3^c^	2.87 ± 0.1^bc^	2.90 ± 0.1^bc^	3.12 ± 0.1^b^	2.67 ± 0.3^c^	3.60 ± 0.1^a^	1.50 ± 0.2^de^
11	Luteolin	0.73 ± 0.1^b^	0.06^d^	0.41^c^	nd	0.31^c^	0.65^b^	0.32^c^	2.52 ± 0.3^a^	0.61 ± 0.1^b^	0.31^c^
12	Apigenin 7-*O*-rutinoside	3.39 ± 0.5^b^	nd	nd	nd	nd	nd	nd	nd	nd	5.16 ± 0.4^a^
13	Apigenin 7-*O*-glucoside	1.52 ± 0.1^c^	nd	nd	3.36 ± 0.4^a^	nd	nd	nd	2.69 ± 0.4^b^	2.69 ± 0.1^b^	0.71 ± 0.1^d^
14	Apigenin 7-*O*-glucuronide	4.41 ± 0.2	nd	nd	nd	nd	nd	nd	nd	nd	4.20 ± 0.5
15	Apigenin malonylglucoside	3.44 ± 0.2^a^	0.19^f^	1.08 ± 0.1^d^	1.25 ± 0.1^cd^	0.66 ± 0.1^e^	nd	1.24 ± 0.1^cd^	1.46 ± 0.2^c^	1.85 ± 0.2^b^	0.63 ± 0.1^e^
16	Apigenin	0.20^a^	trace	nd	nd	trace	trace	trace	0.24^a^	trace	0.15^b^
17	Narirutin	nd	0.54^c^	nd	0.51^c^	0.56^bc^	nd	0.58^bc^	nd	0.65^ab^	0.71 ± 0.1^a^

*Different letters indicate statistical significance at P ≤ 0.05. Each value represents the mean ± standard deviation (n = 3)*.

*nd, not detected*.

**Table 6:** **Phenolic content (g kg^−1^ DM) of the leaves of plants grown in 2011**.

**No**.	**Compound**	**Genotype**									
		**Altilis 41**	**Romanesco C_3_**	**1**	**12**	**35**	**48**	**69**	**72**	**74**	**78**
1	1-*O*-caffeoylquinic acid	nd^(b)^	nd	nd	nd	nd	nd	nd	0.01	0.01	nd
2	3-*O*-caffeoylquinic acid	nd	nd	nd	trace	nd	0.28	nd	trace	trace	nd
3	5-*O*-caffeoylquinic acid	0.86^f^	0.16^h^	0.27^gh^	0.63 ± 0.1^fg^	1.41 ± 0.2^e^	2.83 ± 0.3^d^	4.53 ± 0.7^b^	1.83^e^	3.73 ± 0.3^c^	7.28 ± 0.2^a^
4	1,5-*O*-dicaffeoylquinic acid	0.22^f^	0.28^ef^	0.19^f^	0.46 ± 0.1^ef^	0.52 ± 0.1^e^	3.11 ± 0.5^b^	0.99 ± 0.1^d^	1.82^c^	1.00 ± 0.1^d^	3.72 ± 0.1^a^
5	monosuccinyldicaffeoylquinic acid	nd	nd	nd	nd	nd	trace	nd	nd	0.07	nd
6	4,5-*O*-dicaffeoylquinic acid	nd	nd	nd	nd	nd	trace	nd	0.01^b^	0.12^a^	nd
7	Luteolin 7-*O*-rutinoside	nd	nd	nd	nd	nd	nd	nd	nd	nd	nd
8	Luteolin 7-*O*-glucoside	nd	1.45 ± 0.1^d^	1.70 ± 0.1^c^	1.85 ± 0.3^c^	nd	nd	nd	nd	4.75 ± 0.1^a^	4.22 ± 0.2^b^
9	Luteolin 7-*O*-glucuronide	1.49 ± 0.1^e^	1.64 ± 0.1^e^	1.58 ± 0.1^e^	2.91 ± 0.4^bc^	2.53 ± 0.2^cd^	4.05 ± 0.5^a^	4.29 ± 0.6^a^	3.41 ± 0.1^b^	nd	2.35 ± 0.1^d^
10	Luteolin 7-*O*-malonylglucoside	0.59^g^	0.74 ± 0.1^fg^	0.98 ± 0.1^ef^	1.21 ± 0.2^de^	1.31 ± 0.1^d^	1.91 ± 0.3^bc^	1.98 ± 0.3^b^	1.68^c^	3.31 ± 0.1^a^	1.10 ± 0.2^de^
11	Luteolin	0.19^d^	0.26^c^	0.38 ± 0.1^b^	trace	0.47 ± 0.1^a^	0.22^cd^	trace	trace	0.23^cd^	trace
12	Apigenin 7-*O*-rutinoside	nd	nd	nd	nd	nd	nd	nd	nd	nd	nd
13	Apigenin 7-*O*-glucoside	0.63^a^	0.21^b^	0.55^a^	trace	nd	0.69 ± 0.2^a^	nd	trace	trace	trace
14	Apigenin 7-*O*-glucuronide	0.47^d^	0.45^d^	0.39^d^	0.62 ± 0.1^c^	trace	0.46 ± 0.1^d^	1.09 ± 0.2^a^	0.37^d^	0.84 ± 0.1^b^	0.80^b^
15	Apigenin malonylglucoside	0.45^b^	0.28^d^	0.28^d^	trace	trace	trace	0.46 ± 0.1^ab^	0.38^c^	0.52 ± 0.1^a^	nd
16	Apigenin	nd	nd	nd	nd	nd	nd	nd	nd	nd	nd
17	Narirutin	0.24^d^	trace	0.25^cd^	nd	trace	0.34^ab^	0.39 ± 0.1^a^	0.26^cd^	0.31^bc^	nd

*Different letters indicate statistical significance at P ≤ 0.05. Each value represents the mean ± standard deviation (n = 3)*.

*nd, not detected*.

The most abundant of the luteolin and apigenin derivatives were, respectively, luteolin 7-*O*-glucoside and apigenin malonyl-glucoside. The leaves of selection 69 harbored the highest content in narirutin, apigenin malonylglucoside, apigenin-7-*O*-glucuronide, and luteolin-7-*O*-glucuronide in 2011 (0.39, 0.46, 1.09, and 4.29 g kg^−1^ DM). It has been noted in potato that although the quantity of polyphenols produced by a given genotype may vary from season to season or from locality to locality, the identity of the compounds present in general does not (Andrè et al., 2009). “Altilis 41” leaf contained more of the apigenin derivatives than any of the other genotypes in both 2010 and 2011, and there was no transgressive segregation for the content of apigenin derivatives among the F1 progeny. Genotype × environment interactions can often represent a highly important determinant of the phenotypic performance of lines, a problem which confronts crop breeding programmes which seek to identify germplasm which is as widely adapted as possible. With respect to TMP, transgressive segregation ensured that a number of the F_1_ progeny out-performed the parental genotypes in both seasons (Table 2). The less favorable environmental conditions experienced in 2011 strongly reduced TMP by up to a third in selections 1 and 12, and by a quarter in selection 35. However, selections 74 and 78 were less environmentally labile, implying that it should be possible to breed for *C. cardunculus* germplasm which performs stably with respect to TMP.

### Genotypic variation among the F_1_ progeny

**Figure 4:**
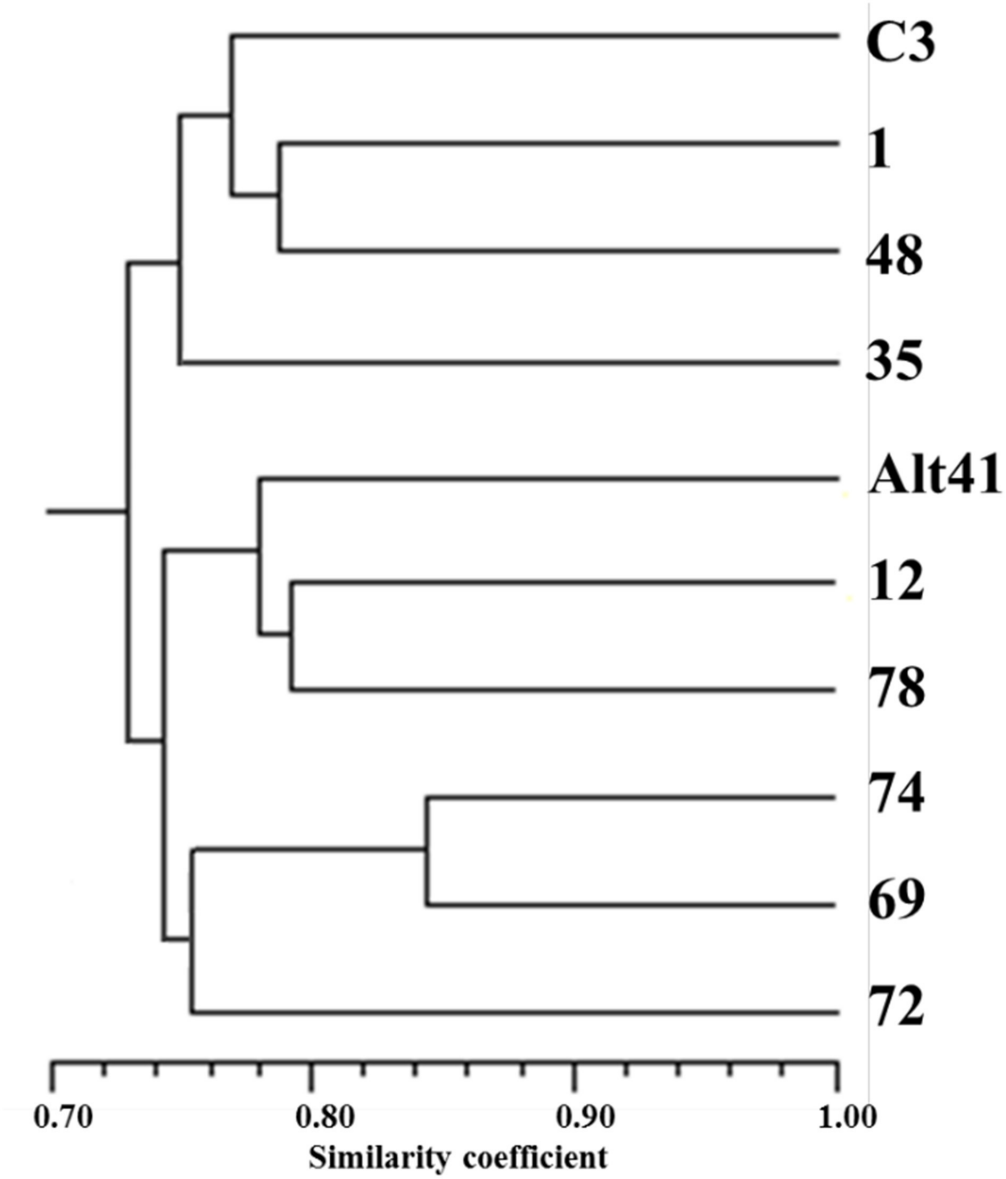
**UPGMA-based cluster analysis**. Cluster analysis has been performed using the SSR genotype (217 loci) of “C3” (“Romanesco C3”), A41 (“Altilis 41”) and eight selected F_1_ segregants derived from the cross “Romanesco C3” × “Altilis 41.”

Out of the set of 217 SSR loci assayed, 59 segregated in a manner consistent with a 1:1:1:1 ratio; of these, 29 involved the segregation of four alleles (both parents being heterozygous for distinct alleles), while the other 30 involved the segregation of three alleles (one parent having the genotype *ab* and the other *ac*). Eight loci segregated 1:2:1 (both parents having the genotype *ab*) and at the remaining 150 loci, the ratio was 1:1 (one parent having the genotype *ab* and the other *aa*). The 372 alleles recognized were used to elaborate a phylogeny (Figure 4), in which two major clades were formed, each including one of the two parental genotypes. The most similar pair of individuals (selections 74 and 69) shared 84% of their alleles. Lanteri et al. (2012) have shown that the progeny of the “Romanesco C3” × “Altilis 41” cross can be fully discriminated using a set of nine SSRs, each mapping to a different chromosome (Portis et al., 2009). The minimum number of SSR loci needed to fully discriminate between the eight selected F_1_ progeny was just four (Table 7). Interestingly the similarity matrix based on the allelic constitution at these four SSRs among the eight progeny was moderately congruent (*r* = 0.43) with its equivalent based on the full set of 217 SSRs (*r* = 0.43).

**Table 7:** **The allelic status at the four SSR loci required to fully discriminate between all eight selected F_1_ progeny (“C3”: “Romanesco C3,” “Alt 41”: “Altilis 41”)**.

	CELMS-23 (LG 12)^*^	**CELMS-58 (LG 1)**	**CELMS-60 (LG 9)**	**CMAL-21 (LG 13)**
C3	ab	ab	ab	ab
Alt41	cd	cd	cd	cd
1	bd	ac	bc	bc
12	ad	bd	bd	bc
35	ac	bc	ac	ac
48	bc	bc	bd	ad
69	ad	bd	ad	ad
72	bc	ac	bc	bd
74	ad	bd	ad	bc
78	ad	bd	bd	ac

* *The mapping position of each SSR is reported in brackets*.

As reported by Lanteri et al. (2012), it was possible to fingerprint each individual of the “Romanesco C3” × “Altilis 41” progeny by applying a set of 9 SSRs (i.e., CELMS-01, -15, -23, -24, -37, -41, -58, -60, and CMAL-21) which are dispersed across the linkage groups (Portis et al., 2009, see Supplementary Figure 1) and displayed a four allele segregation.

## Conclusions

Profiling polyphenols among the F_1_ segregants from the cross var. *scolymus* × var. *altilis* succeeded in identifying eight transgressive individuals with respect to TMP content. Selections 48 and 78 were of particular interest as characterized by an high biomass production and their accumulation of CQAs was clearly less environmentally labile than that of the other genotypes and the parents. The implication is that there is indeed potential for breeding *C. cardunculus* as a source of compounds of pharmaceutical and nutraceutical interest. A particular advantage of this crop is that any desirable genotype can be easily vegetatively propagated, either using actively growing or semi-dormant shoots (the latter develop on the underground stem); as an alternative, it has also been shown that plants can be regenerated by *in vitro* micropropagation. A set of four SSR loci, evenly distributed across the genome is sufficient to unambiguously identify each of the eight transgressive genotypes. The genetic identity of such vegetatively propagated materials can thus be readily established using SSR-based profiling.

## Author contributions

Sergio Lanteri and Giovanni Mauromicale designed and planned the experiments. Gaetano Pandino and Sara Lombardo undertook the crop management and plant sampling, including the HPLC polyphenols profile. Andrea Moglia undertook HPLC preliminary analysis in F_1_ population. Ezio Portis undertook SSR-based fingerprinting analyses. All the authors drafted the manuscript and approved its final version.

### Conflict of interest statement

The authors declare that the research was conducted in the absence of any commercial or financial relationships that could be construed as a potential conflict of interest.

## Supplementary material

Supplementary Figure 1 **The SSR-based consensus map of *C. cardunculus***. Marker names are shown to the right of each LG, with map distances (in cM) to the left. “r-“ and “a-“ indicate markers segregating only in, respectively, “Romanesco C3” and “Altilis 41.” Segments shaded in red indicate where a pair of LGs has merged as a result of reducing the stringency to LOD 5. The nine loci needed to obtain specific molecular fingerprinting for each individual of the progeny have been highlighted in red.

## Abbreviations

CQA: caffeoylquinic acids
CGA: chlorogenic acid.
